# Engineered Phage-Guided Nanotherapeutic Systems for Precision Antibacterial Therapy: Hacking Bacterial Resistance Mechanisms

**DOI:** 10.3390/pharmaceutics17101288

**Published:** 2025-10-02

**Authors:** Bandar Aldhubiab, Rashed M. Almuqbil

**Affiliations:** Department of Pharmaceutical Sciences, College of Clinical Pharmacy, King Faisal University, Al-Ahsa 31982, Saudi Arabia; ralmuqbil@kfu.edu.sa

**Keywords:** phages, nanotherapeutic systems, antibiotics, resistance, encapsulation, bacterial biofilms

## Abstract

Antibiotic resistance (ABR) poses a critical global public health challenge necessitating immediate action. Without prompt interventions, infections caused by antibiotic-resistant bacteria could surpass the annual mortality rates of all cancers combined by 2050. Phages are one of the most abundant biological entities on earth that specifically infect and replicate in bacterial cells and can act as potential alternatives to antibiotics. Nanotechnology provides a favorable solution to overcome various challenges linked with phage therapy. Developments in nanotechnology, including nano-encapsulation, offer solutions to various clinical as well as pharmacological challenges by improving delivery efficacy, ensuring controlled release, and protecting phages from environmental degradation and immune clearance. The synergistic actions of phage-guided targeting and the strong bactericidal potential of engineered nanocapsules (NCs) could effectively eradicate multidrug-resistant (MDR) bacteria while diminishing off-target activities. Potential applications of engineered phage-guided nanotherapeutic systems have already been explored in terms of phage/nanocarrier cocktails, enhanced antibacterial activity, effective treatment of nosocomial infections, wound healing, and disruption of bacterial biofilms. The present review focuses on comprehensively discussing the advances in phage-guided NCs along with their mechanisms in enhancing precision antibacterial therapy. In this regard, numerous in vitro and in vivo study findings have been summarized in this review. Moreover, various approaches to overcome and optimize the pharmacokinetic profiles of phage-guided NCs have been discussed.

## 1. Introduction

Antibiotic resistance (ABR) in bacteria is a global threat to public health that requires urgent attention [1,2]. In 2019 alone, ABR was directly responsible for 1.27 million deaths globally and was linked with around 5 million deaths [3]. Multidrug-resistant (MDR) bacteria, predominantly those with biofilm-forming capacities, pose a significant danger as they have the ability to cause recurrent and chronic infections [4]. Bacterial biofilms are complex communities of microorganisms encased in extracellular polymeric substances. These biofilms show heightened resistance to antibiotics and various other antimicrobial therapies [5]. Biofilms can also mediate horizontal gene transfer, which can result in the spread of ABR genes [6]. In addition, biofilms facilitate microbial populations to colonize both animal and human hosts, as well as allow their attachment to inert surfaces. It has been observed that these characteristics are contributing to the growing occurrence of infections associated with the usage of biomaterials in both veterinary and human medicine [7]. The lack of novel antibiotics threatens our ability to control resistant strains, and projections indicate ABR-related deaths may surpass cancer mortality by 2050 [8]. Therefore, there is a critical need for the development of alternative approaches to manage biofilm-forming MDR infections and prevent the advancement of ABR [9].

Bacteriophages, commonly known as phages, are among the most prevalent biological entities on Earth [10]. These viruses specifically target and replicate within bacterial cells. They have the privilege to act as potential alternatives to antibiotics. Phages were independently discovered by Frederick Twort and Felix d’Hérelle in 1915 and 1917, respectively [11]. The increasing MDR cases have revived the research on phage therapy in the 21st century. A number of studies have already demonstrated the significance of phage therapy in the treatment of various types of infections, including pneumonia [12], osteomyelitis, urinary tract infection [13], and sepsis [14]. Phage production methods are cost-effective, swift, and straightforward due to the self-replicating nature of phages; however, this advantage is contingent upon the specific bacterial host species [15]. Despite the promising potential of phage therapy, it has not received widespread regulatory approval for human use. Antibiotics often provide a broad-spectrum activity, thus resulting in indiscriminate killing or suppression of various beneficial microbiota [16]. On the other hand, phages precisely target and attack designated bacterial hosts (Table 1).

**Figure 1:**
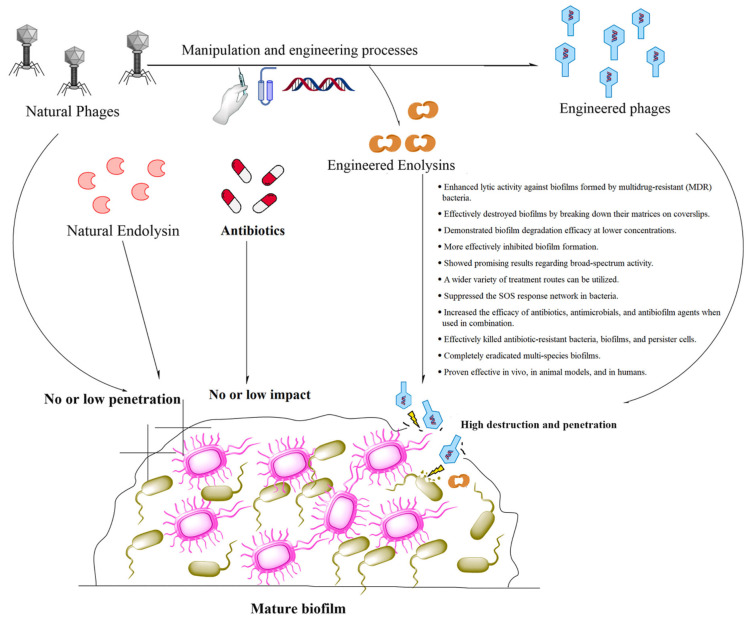
Potential antibiofilm strategies based on engineered phages. Reproduced from Reference [38]. Copyright 2024, Elsevier B.V. Biofilms are aggregates of antibiotic-resistant bacteria, which are often difficult to penetrate and eliminate because of their lack of response or low penetrability to antibiotics, natural phages, and endolysins. It has been reported that targeted manipulation or engineering of endolysins and phages can result in enhancing their capacities to penetrate as well as eradicate biofilms and abolish antibiotic-resistant bacteria.

Nanotechnology provides a more favorable solution to overcome various challenges linked with phage preparations. Developments in nanotechnology, including nano-encapsulation, offer solutions to various clinical as well as pharmacological challenges by improving delivery efficacy, ensuring controlled release, and protecting phages from environmental degradation and immune clearance [33]. Encapsulation of phages within nanovesicles can protect phages and provide a controlled release [34]. Nanofibers produced via electrospinning are an alternative and promising method to improve the outcomes of phage therapy [33]. Integration of nanotechnology with phage therapy offers several benefits, including overcoming the limitations of phage-only formulations, ensuring better therapeutic efficiency, and optimizing the pharmacokinetic profile of phages. Phages are also used as cocktails with antibiotics that offer several advantages [35]. Phages can disturb bacterial biofilms and enhance antibiotic penetration as well as efficiency [36], whereas antibiotics can diminish bacterial defenses and make them more prone to phage attacks [37]. Moreover, researchers have developed novel antimicrobial agents via the alteration of phages and endolysins to overcome the phage therapy-associated challenges. These newly developed agents can selectively target bacteria and eliminate biofilms, which is a novel approach to tackle ABR infections and biofilms (Figure 1) [38].

This review comprehensively examines advancements in phage-guided nanocarriers (NCs) and their mechanisms for improving precision antibacterial therapy. It summarizes findings from numerous in vitro and in vivo studies. Additionally, strategies to enhance and optimize the pharmacokinetic profiles of phage-guided nanotherapeutic systems are explored.

## 2. Engineering Phage-Guided Nanocarriers to Enable Precise Pathogen Targeting

**Figure 2:**
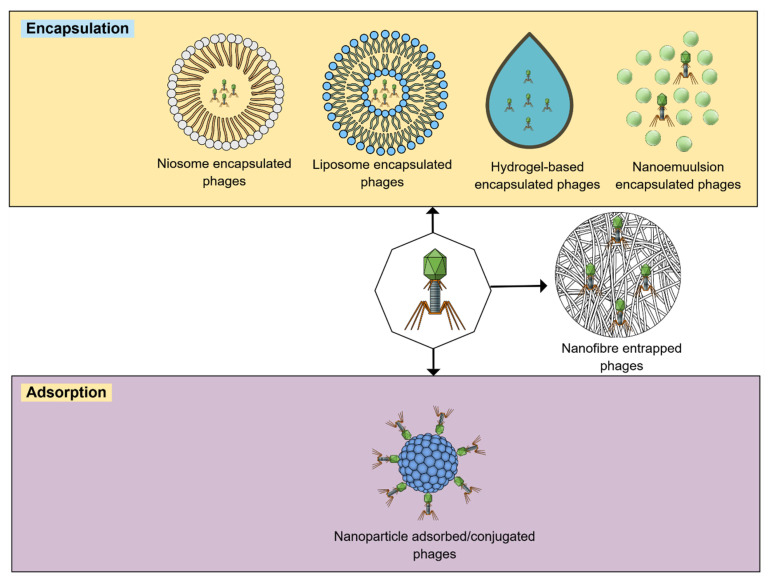
Multiple potential phage delivery strategies. These strategies protect phages from physicochemical stresses and/or immunological reactions.

In recent years, nanotechnology has gained traction as a novel approach in healthcare, offering potential alternatives to antibiotics and traditional phage therapy. NCs are colloidal drug carrier systems having sizes less than 500 nm. NCs are revolutionizing drug delivery systems by encapsulating and delivering therapeutic drugs by using nanosized vehicles to improve bioavailability, drug efficacy, as well as controlled, targeted, and specific drug release [39]. Most commonly used and potential NCs include lipid-based NCs, micro- and nanoemulsions, exosomes, dendrimers, niosomes, and liposomes [40,41,42,43]. Furthermore, the development of new antimicrobial agents is mediated by various engineered nanomaterial designs including nanoscale metal–organic frameworks, polymeric NCs, nanospheres, mesoporous silica nanoparticles, and carbon nanotubes [44,45,46,47]. A range of delivery and encapsulation techniques has already been implemented to enhance the intracellular delivery and stability of phages (Figure 2).

The population dynamics of phage-bacteria are crucial for the success of phage-based therapies in the eradication of infections caused by bacteria [33]. Higher densities of phages are required to inhibit the growth of bacteria that are susceptible to phage therapy; however, continuous phage amplification is also important to limit the multiplication as well as spread of bacteria. However, phage amplification relies on the bacterial concentration; thus, a minimum level of bacterial concentration is required at the target site [48]. On the other hand, if phage is administered at earlier phases of infection or if a lower level of bacterial density is present, bacteria might take some time for their growth before achieving the thresholds to mediate effective amplification of phages [25]. In these cases, phages might decline in initial titer or face earlier decay. In addition, there are various other factors that can cause clearance of phages including enzymes, immune factors of the host, and various other stress factors or tissue fluids, which can further reduce viable titer, leading to reduced effectiveness of phage therapies [20,49]. In such scenarios, the encapsulation process can be an efficient and potentially useful approach that can serve in various applications. The advantages of encapsulation include prolonged release of viable phages over an extended period, playing a role as an active delivery system, and shielding or protecting phages from the outer environment [50,51,52]. Therefore, the encapsulation process helps in maintaining titers of phages to therapeutically efficient concentrations over an extended period in a way that once the threshold level of bacteria is achieved, titers of phages can further amplify, resulting in effective and faster action [25,51,53]. Furthermore, substantial advances have been achieved in addressing various challenges linked with phage therapy by using modern nanoscale-based encapsulation approaches [33].

### 2.1. Encapsulation

Modern lipid-based nanovesicle is a type of NC that offers versatile and promising drug delivery approaches involving vesicles composed of synthetic or natural lipids. It has been reported that these NCs self-enclose and self-assemble to generate spheres composed of lipid bilayers containing inner aqueous cores that have the potential to encapsulate and protect sensitive drugs [54]. Liposomes are a type of lipid-based nanovesicle that have excellent biocompatibility with phages without disturbing their numbers and efficacy both during as well as after encapsulation [55,56]. The use of liposomes was found to be beneficial in phage delivery in order to target biofilm-associated pathogens, intracellular pathogens, gut pathogens, and so on. These non-immunogenic and non-toxic NCs also provide protection to phages against various outer stress factors, including elimination from the reticuloendothelial system, highly acidic pH of the gastric system during gastrointestinal transit, and functions of neutralizing antibodies, henceforth improving the circulation time in vivo [55,57,58,59,60]. The preparation method of liposomes is easy, and the encapsulation process can effectively control a range of physical factors, including encapsulation dose, encapsulating efficiency, and vesicle size. Liposomes have the capacity to mimic biological membranes with regard to fluidity, behavior, and structure. Therefore, they can go through multiple dynamic and conformational transitions that are crucial for numerous biological actions. In this regard, for instance, liposomes can cross several host tissue barrier layers, including deep-seated tissues, intestinal mucosa, and epidermal layers, as well as bacterial mature biofilms, facilitating deeper delivery of the entrapped drugs to such areas [33,61].

Liposome complexation is a technique that can be used to modify the structure of a liposome to ameliorate its pharmacokinetic profile, stability, and physicochemical properties. Liposomes that have been complexed are commonly referred to as liposome complexes. Polymers can be used as a complex-forming agent. It has been observed that each polymer type is likely to cause different types of complex shapes as well as interactions with liposomes [62].

Other particle production techniques for phage encapsulation involve spray drying, electrospray, and freeze drying. Lyophilization or freeze drying method is widely used to preserve proteinaceous compounds, which can also be used to prepare dried particles with phages [63]. The manufacturing method of lyophilized phage particles is simple, which includes freezing a solution containing phages at −20 °C and then removing water through sublimation and desorption under vacuum conditions [64].

Various studies have already encapsulated phages in various polymers and liposomes. For example, in a study, Singla et al. [58] used a conventional approach to prepare cationic liposome-encapsulated phages in order to ensure efficient intracellular delivery into macrophages infected by *Klebsiella pneumoniae* (*K. pneumoniae*). The researchers killed any extracellular bacteria by using gentamicin in the culture media, since their focus was on intracellular bacteria only [58]. Moreover, it was observed that liposome-encapsulated phages were protected against neutralizing antibodies in comparison with the free phages [58]. In a different study, Chadha et al. encapsulated a phage cocktail in liposomes [56]. The developed liposome-loaded phage cocktail showed improved therapeutic efficacy against *K. pneumoniae* infection in burn wounds in BALB/c mice [58]. The average size of the liposome-loaded phages was 229 nm, which exhibited 79.2% ± 5.6 encapsulation efficiency. Following 3 days of administration, liposome-encapsulated phage cocktail exhibited 1–2 log order decrease in bacterial counts in comparison with the free phages in liver, blood, and skin [58]. In another study, Rios et al. [65] used liposomes as a vehicle for phage delivery. The encapsulation of phages in liposomes provided 100% protection to the phage against neutralizing antibodies. In contrast, the unentrapped phages were neutralized by the neutralizing antibodies within 3 h of their interactions. The researchers found that free phages could not penetrate macrophages, whereas liposome-encapsulated phages successfully delivered their payload into macrophages, achieving a 94.6% reduction in intracellular *K. pneumoniae*. Furthermore, these liposome-encapsulated phages demonstrated synergistic effects with amikacin, effectively eradicating mature *K. pneumoniae* biofilms.

Despite considerable research, no significant progress has been reported in terms of clinical translation of liposome-assisted drug delivery platforms owing to several challenges. These challenges include cost, multiple chemical synthesis steps and formulation processes, difficulty in large-scale good manufacturing production, and presence of increased number of complex physicochemical variables [66,67].

Niosome-encapsulated endolysins have also exhibited several beneficial effects. In a study, Marchianò et al. [68] successfully encapsulated antistaphylococcal lytic protein (CHAPSH3b) in non-ionic niosomes via combining cetyltrimethylammonium bromide, cholesterol, and Span 60 at concentrations that warranted the desired vesicle size. The researchers observed a synergistic effect between cetyltrimethylammonium bromide and phage protein against planktonic bacteria even after 2 weeks of storage. On the other hand, phage-based hydrogels show good biocompatibility as well as antibacterial action. Phage-based hydrogels exert excellent antibacterial actions on targeted bacteria owing to their specific targeted bactericidal activity; however, only a few studies have explored the antibacterial actions of phage hydrogels so far [69]. In a study, Abed et al. [70] fabricated phage-based hydrogels by utilizing hyaluronic acid, carboxymethyl cellulose, and sodium alginate to treat wounds infected by *Enterococcus faecalis*. The developed phage-containing hydrogels efficiently treated the infected wounds and mediated wound healing through controlled phage release [70].

Emulsification is an important encapsulation technique, where uniform emulsions can be prepared by mixing drugs, enzymes, cells, and microbes with a well-suited polymer, and this mixture can be further dispersed into another phase of vegetable oils [71]. In addition, the prepared emulsions can be further stabilized by adding stabilizing agents and emulsifiers. Therefore, nanoemulsions provide a new approach to encapsulate various sensitive molecules, including phages, enzymes, and proteins, in a nanoporous matrix. It has been observed that the entrapment of bio-molecules within nanoemulsions results in an alteration in the surrounding water activity, which provides increased stability during storage [72]. Moreover, preparation of such emulsion formulations can provide protection to the phages against inactivation caused by outer proteolytic enzymes and the immune system, while preserving their structures and functions [33]. However, hydrophile–lipophile balance (HLB) must be carefully considered while choosing an emulsifier. It has been observed that emulsifiers with high HLB values (8–18) are best suited for oil-in-water nanoemulsions, whereas emulsifiers with low HLB values (3–6) are best-suited for water-in-oil nanoemulsions [73]. Nanoemulsions provide a highly uniform droplet size, which improves reproducibility and controlled drug release [74]. However, several challenges might be encountered while working with nanoemulsions. For example, the use of a large amount of surfactant and co-surfactant sometimes makes this delivery system bulky and toxic. It has also been reported that nanoemulsions prepared by phase inversion techniques show susceptibility to flocculation and Ostwald ripening [75].

### 2.2. Nanofibers

Nanofibers are typically produced via electrospinning, a process that uses a strong electric field to transform polymer solutions into continuous nanoscale fibers [76]. In electrospinning, a polymer solution is extruded through a syringe needle, forming a droplet. A high-voltage power supply connects the needle and collector, generating an electric field between them. This field induces surface charges on the droplet, causing it to elongate into a conical shape. Subsequently, a jet of polymer solution emerges from the cone, stretching toward the oppositely charged collector. During this trajectory, the solvent evaporates, and the polymer solidifies, forming continuous nanofibers on the collector surface [77]. Phage-incorporated nanofibers have been developed to combat pathogenic bacteria [76]. For example, Nogueira et al. [78] immobilized vB_Pae_Kakheti25 phage capsids onto polycaprolactone (PCL) nanofibers, creating a laundry-resistant, antimicrobial, and non-toxic dressing for biomedical applications. Surface analysis confirmed that the phage capsids were oriented on the PCL nanofibers to optimize tail-mediated bacterial interactions. Antimicrobial assays demonstrated a highly effective 6-log bacterial reduction (99.9999%) upon immediate contact and after 2 h, even following 25 washing cycles.

### 2.3. Stimuli-Responsive Nanocarriers

**Figure 3:**
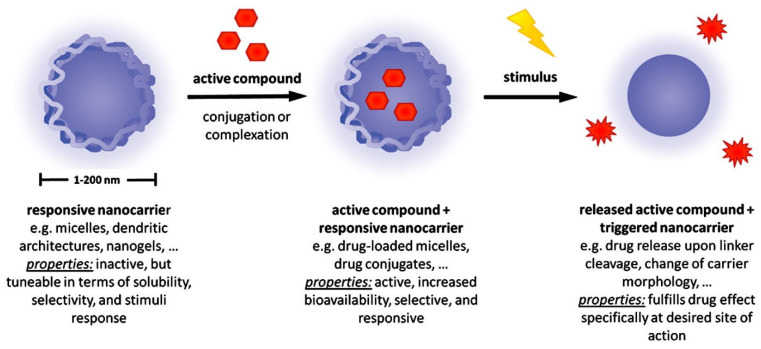
Schematic diagram of a stimuli-responsive nanocarrier for the delivery of active compounds. Reproduced with permission from Reference [86]. Copyright 2012, Elsevier B.V.

In terms of drug delivery, there is a growing interest in the smart release platforms because of their capacity to release active compounds or drugs at the targeted area in response to external stimuli (Figure 3). Stimuli-responsive nanoplatforms have the capacity to respond to various physical stimuli (for example, magnetic fields, ultrasound, light) and changes in the infection microenvironment (for example, enzymes, pH, hydrogen peroxide) [79,80]. Indeed, these stimuli-responsive antibacterial nanomaterials can be cleverly designed to maximize the antibacterial effects and minimize toxic side effects [81]. Eudragits are synthetic polymers produced by polymerization of acrylic acid as well as methacrylic acid or their esters, such as dimethylaminoethyl ester or butyl ester [82]. Eudragit polymers are polymethacrylates that act as pH-responsive polymers by reacting to alterations in pH [33,83]. There are different grades of Eudragit, for instance, Eudragit S100 dissolves at pH 7, while Eudragit L100 dissolves at a pH of 6. Therefore, the usage of such polymers in the encapsulation matrix enables drug release solely at the desired site and at the correct time, upon reaching the desired pH. On the other hand, Eudragit S and Eudragit L are two highly stable forms that can generate coatings or films resistant against low gastric pH, thus mediating drugs to effortlessly circumvent the stomach and reach the intestine [84]. In addition to this, these films are soluble in intestinal fluid at pH 6 and 7, where they secrete the active compound [33,85]. In a similar manner, phages can be encapsulated and protected against gastric pH, which will allow phage release only at the desired area upon reaching the desired pH. This approach can be beneficial for the success of oral phage therapy against a range of enteric infections [33]. Various studies have already developed formulations based on Eudragit microparticles and microspheres to enhance the stability of phages.

In a study, Vinner et al. [87] encapsulated *myovirus* CDKM9, a *Clostridium difficile*-specific phage, in Eudragit S100 (with and without alginate) by utilizing microfluidic glass capillary devices for colon delivery using pH-triggered release. The researchers trapped phage-containing highly monodispersed core–shell microparticles within the particle core, which were prepared by in situ polymer curing by utilizing 4-aminobenzoic acid dissolved in the oil phase. As compared to free phages, the encapsulated phages within the microparticles showed resistance against simulated gastric fluid at pH 2, mimicking the human stomach environment, for 3 h, and then showed a pH-triggered burst release at pH 7 [87]. In a different study, Vinner et al. [88] developed microencapsulated *Escherichia coli* (*E. coli*)-phages in a pH-responsive solid oral dosage formulation. They produced Eudragit S100 and alginate containing uniform pH-responsive composite microparticles. The microencapsulation technique provided protection to phages when exposed to a simulated gastric acidic environment for a prolonged period. In addition, the encapsulated phages pre-exposed to simulated gastric acid were then added to actively growing bacterial cells by utilizing in vitro cell cultures, and the phages showed effective killing of *E. coli*. The researchers also observed through confocal microscopy that the morphology of encapsulated phage-receiving epithelial cells was significantly better compared to controls without phage therapy. Moreover, the encapsulated phages were found to be stable when stored over a four-week period in a refrigerator [88].

Poly(N-isopropylacrylamide) (PNIPAM) is a thermosensitive polymer that undergoes phase transition in response to temperature variations [89]. In a study, Hathaway et al. [90] developed poly(N-isopropylacrylamide-co-allylamine) (PNIPAM-co-ALA) nanospheres for temperature-triggered release of phage K. These nanospheres, leveraging the thermosensitive properties of the polymer, were designed to collapse at elevated temperatures associated with bacterial skin infections. The phage-loaded PNIPAM-co-ALA nanospheres effectively lysed *Staphylococcus aureus* ST228 at 37 °C, while bacterial growth remained unaffected at 25 °C, demonstrating precise thermally induced phage release. In another study, Hathaway et al. [91] utilized PNIPAM to control methicillin-resistant *Staphylococcus aureus* (MRSA) by enabling the temperature-dependent release of a synergistic enzybiotic cocktail containing staphylococcal bacteriocin lysostaphin and truncated phage endolysin CHAPK. Bacterial lysis was observed at 37 °C, whereas bacterial growth persisted at the uninfected skin temperature of 32 °C [91].

Despite promising progress in stimuli-responsive NCs, their widespread clinical translation still faces critical challenges. Major challenges for clinical translations include reproducibility in manufacturing, cost, and scalability. Laboratory-scale formulation and synthesis techniques are good enough for proof-of-concept studies; however, they often encounter substantial challenges during scale-up to industrial production. At industrial scales, it is very difficult to ensure batch-to-batch consistency in terms of release kinetics, drug loading efficiency, surface characteristics, and size distribution [92].

## 3. Mechanisms by Which Phage-Guided Nanocarriers Accurately Combat Bacterial Pathogens

**Figure 4:**
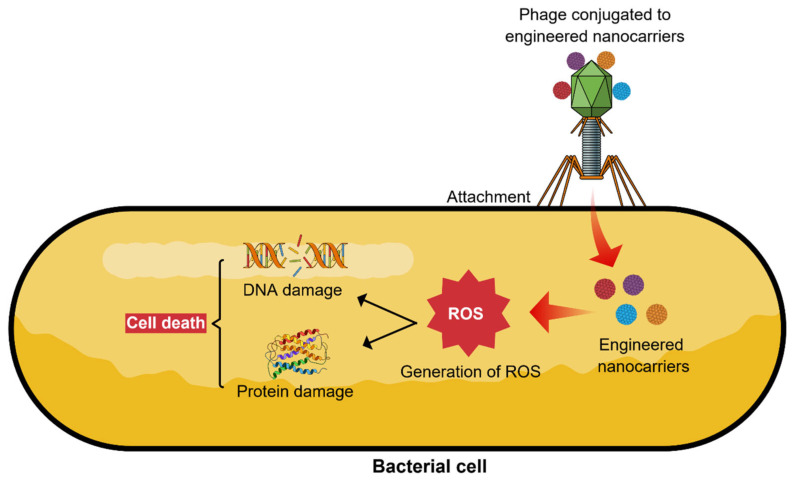
Antimicrobial mechanism of phage-guided nanocarriers. The engineered nanocarriers conjugated to phages disrupt the cell membranes of bacteria. Then, the nanocarriers produce toxic reactive oxygen species (ROS) and induce various intracellular effects, including DNA and protein damage, which eventually result in cell death.

In order to exert antimicrobial activities, an agent needs to gain access to the molecular targets that are associated with the metabolism of microbial cells, including protein synthesis and cell wall synthesis [93]. The integration of phages with engineered NCs is a crucial step for the phage-guided NCs that act against pathogenic bacteria. Engineered NCs loaded with various therapeutic agents can be precisely transported to the infected microenvironment. Chemically or naturally engineered NCs can exert powerful antimicrobial actions by using a range of mechanisms that involve reactive oxygen species (ROS) that can interrupt DNA synthesis, disturb enzymatic activity, and damage internal cell organelles [93]. In terms of the mechanism of phage-guided NCs, firstly, phages need to be conjugated with the engineered NCs, which would enhance selective detection and target specificity [94,95,96]. Receptor binding proteins (RBPs) in phages are specialized proteins located at the tail of phages. RBPs are exclusively associated with the host-phage interaction, since they are accountable for the identification of bacterial hosts [52]. On the other hand, cell wall binding domains (CBDs) of phage endolysins have the capacity to target the enzymes to their substrates in the bacterial peptidoglycans along with extraordinary specificity [97]. Following attachment, the phage-guided engineered NCs can fight against the infections by penetrating bacterial cell membranes, generating ROS, DNA damage, and protein damage, which eventually results in bacterial cell death (Figure 4) [51].

A range of studies have already demonstrated the efficacy of various phage-guided engineered NCs. In a study, Peng et al. [98] conjugated engineered phages with gold nanorods for selective attachment with various Gram-negative organisms, such as the human pathogens including *Vibrio cholerae*, *Pseudomonas aeruginosa* (*P. aeruginosa*), and *E. coli*, and the plant pathogen *Xanthomonas campestris*. The researchers reported that the engineered phages selectively delivered gold nanorods to the targeted bacteria, and the nanorods terminated both phages and bacteria simultaneously [98]. In another study, Yan et al. [99] used lysin CBD derived from a novel virulent MRSA phage Z for gold nanosheet functionalization. The researchers confirmed that the phage lysin-CBD-modified gold nanosheets potentially delivered directly to MRSA and effectively eradicated them under near-infrared irradiation in vitro, while exhibiting satisfactory in vivo biocompatibility [99]. Collectively, the findings suggest the synergistic actions of phage-guided targeting and strong bactericidal potential of engineered NCs, which could effectively eradicate MDR bacteria while diminishing off-target activities [96].

## 4. Potential Applications of Engineered Phage-Guided Nanotherapeutic Systems for Precision Antibacterial Therapy

### 4.1. Management of Nosocomial Infections

ABR is an increasing and life-threatening public health concern, particularly in surgical patients [57,58]. In addition, ABR further creates complications in nosocomial infections by elevating costs, morbidity, and mortality. The Centers for Disease Control and Prevention (CDC) listed a range of bacteria that are responsible for causing severe infections in humans and are responsible for the highest risk of developing multidrug resistance. Unfortunately, most of these bacteria are commonly found in hospitals/hospital facilities, spreading through human contact and surfaces. The 6 highly drug-resistant bacteria are known as ESKAPE pathogens (*Enterococcus faecium*, *Staphylococcus aureus*, *Klebsiella pneumoniae*, *Acinetobacter baumannii* (*A. baumannii*), *P. aeruginosa*, and *Enterobacter* species), causing hospital-acquired infections or nosocomial infections worldwide and progressively showing multidrug resistance [100]. Over two-thirds of nosocomial infections are likely to be turned into high-density biofilm, skin, or chronic infections, which are often troublesome to treat because of their adaptive nature and virulence [101]. Various innovative approaches have been adopted in order to tackle such infections, including denaturalizing the lipopolysaccharides present in the aforementioned bacteria. Among them, a potential approach is the use of NCs and endolysins or lysins encoded by phages [102,103].

Silver NCs are extensively utilized in biomedical fields because of their wide range of applications. Researchers have demonstrated the powerful antimicrobial activity of green-synthesized silver NCs against antibiotic-resistant, Gram-positive, and Gram-negative bacteria [104,105,106]. In a study, Ramírez Saenz et al. [107] conjugated BK510 commercial endolysins with metallic NCs to abolish the significant challenges caused by the outer membrane of Gram-negative bacteria. Silver NCs were prepared by green synthesis, and their average size was 16.06 ± 4.23 nm. The silver NCs and endolysins were utilized to prepare conjugates that were assessed against both Gram-positive and Gram-negative bacteria. The study findings suggested that usage of the conjugates showed a higher inhibitory effect than silver NCs in over 65% of the Gram-negative bacteria [107]. In a different study, Hopf et al. [10] developed an NC-based antibacterial system that structurally mimicked the protein-turret distribution on the head structure of certain phages. The researchers explored the antibacterial potential of various combinations of different materials. They synthesized phage-mimicking antibacterial NCs composed of silver-coated gold nanospheres distributed randomly on a silica core. These phage-mimicking NCs were evaluated for their biocompatibility with human skin keratinocytes and bactericidal capacity against 4 clinically relevant nosocomial pathogens, including *Corynebacterium striatum*, *Enterococcus faecalis*, *P. aeruginosa*, and *Staphylococcus aureus* (*S. aureus*). The researchers observed that the bacterial growth of these 4 bacteria was delayed (by up to 5 h) and suppressed (21% to 90%). In addition, the Gram-positive bacteria were found to be more sensitive to the phage-mimicking NCs. These NCs also did not exert any noticeable cytotoxic activities against human skin keratinocytes [10].

### 4.2. Improved Wound Healing

In humans, *S. aureus* is a common inhabitant of the skin and nasal passages [108]. Skin typically acts as a barrier against progressive infection. Nonetheless, a breach in the skin surface because of a burn, cut, or scratch can provide an entry point for opportunistic bacteria. It has been reported that *S. aureus* is one of the most common pathogens responsible for skin and soft tissue infections [109,110]. This pathogen delays wound healing and also causes systemic infections, including sepsis, endocarditis, and osteomyelitis [111]. There is an urgent need for an alternative approach because of the global emergence and increased occurrence of MRSA. The mortality rate and treatment cost of hospital-acquired MRSA are double those of the methicillin-susceptible counterparts [112]. In a study, Hathaway et al. [90] formulated Poly(N-isopropylacrylamide), a thermally responsive polymer, nanospheres copolymerized with allylamine. The researchers demonstrated that by using this polymer, it is possible to engineer the nanospheres in a way that they will collapse at an increased temperature linked with a skin infection caused by bacteria. The developed phage incorporated Poly(N-isopropylacrylamide) nanospheres copolymerized with allylamine successfully lysed *S. aureus* at 37 °C, while the growth of bacteria remained unchanged at 25 °C, therefore providing a thermally triggered phage release.

In another study, Sarhan and Azzazy [113] developed green wound dressings that exhibited potent antibacterial effects and enhanced wound-healing. Polyvinyl alcohol, honey, and chitosan nanofibers were electrospun and loaded with bee venom, propolis and/or phage against the MDR *P. aeruginosa* and evaluated their cytotoxicity, wound healing, as well as antibacterial capacity. The researchers observed that among different nanofiber formulations, polyvinyl alcohol, honey, chitosan-bee venom/phage showed the highest antibacterial activity against all tested Gram-positive and Gram-negative bacteria and caused nearly complete eradication of *P. aeruginosa*. Furthermore, the cytotoxicity experiment demonstrated improved biocompatibility, and the in vivo experiment showed improved wound-healing [113].

Burned areas are particularly prone to bacterial colonization, where approximately 10% of all burn areas eventually become infected [114]. Such infections can elevate morbidity and mortality of patients, and increase treatment costs because of long-term hospitalization [115]. Thus, phages and NCs have emerged as a potential therapy against the burn-related microbial infections [116]. In a study, Esteban et al. [117] evaluated the antibacterial potential of phage K against *S. aureus* over time, when stabilized and delivered via an oil-in-water nano-emulsion. The researchers observed that the phage-loaded nano-emulsion formulations showed enhanced antibacterial action as compared to freely suspended phage. Furthermore, the phage-loaded nano-emulsions resulted in complete and rapid bacterial death of 3 different strains of *S. aureus* [117].

### 4.3. Enhanced Antibacterial Activity

Mesoporous bioactive glass nanoparticles (MBGNs) are promising multifunctional materials that can be chemically modified to introduce antibacterial properties [118]. Advantages of MBGNs include tunable pore structure, large surface area, and small particle size [119]. In a study, Meng et al. [120] engineered MBGNs by utilizing phages to improve the antibacterial property. The researchers used *Salmonella* Typhimurium-specific phage PFPV25.1, which has the capacity to infect *Salmonella enterica* serovar Typhimurium strain LT2, which was utilized as a model phage to engineer MBGNs. They modified MBGNs with amine groups to improve the affinity between phages and the surfaces of MBGNs. It was observed that *Salmonella* Typhimurium-specific phage PFPV25.1 was effectively bound onto MBGNs surfaces without compromising their bioactivity. As compared to non-functionalized MBGNs, an increased level of phages was found to be bound to amine-functionalized MBGNs. In addition, because of this increased phage binding, amine-functionalized MBGNs showed higher antibacterial properties as compared to phage-bound MBGNs [120].

In a different study, Abdelsattar et al. [121] combined 3 phages (ZCPA5, ZCSS1, and ZCSE6) with 3 green synthesized NCs, including silver-chitosan-NCs, pH-sensitive chitosan-NCs, and propolis-chitosan-NPs to treat *P. aeruginosa*, *Staphylococcus sciuri*, and *Salmonella* Typhimurium. Among the NCs, silver-chitosan-NCs showed higher bactericidal properties in combination with phages. In addition, a substantial killing capacity was exhibited by silver-chitosan-NCs (16.5–30.1 μg/mL) in combination with phages [121].

### 4.4. Disruption of Pathogenic Bacterial Biofilms

Phages in the form of cocktails were found to be effective in reducing biofilm growth on various surfaces [122]. Nonetheless, researchers recently identified that biofilms can regrow after 24 h of phage therapy, which further diminishes the effectiveness of phage therapy [123]. Similar findings were observed in the case of *P. aeruginosa* and *A. baumannii* biofilms [124]. Metal NCs, including gold and silver NCs, have demonstrated efficacy against a range of MDR bacterial strains, such as *Listeria monocytogenes*, *Bacillus cereus*, *A. baumannii*, *Staphylococcus epidermidis*, *S. aureus*, *K. pneumoniae*, *P. aeruginosa*, and *E. coli* [125,126,127]. Moreover, these metal NCs were combined with various antibiotics, including gentamicin, ciprofloxacin, meropenem, ceftazidime, and cefotaxime, to kill *K. pneumoniae* and *E. coli* [128]. In a study, Szymczak and Golec [129] evaluated the efficacy of engineered T7 phages armed with silver NCs to eradicate the biofilms of *Escherichia coli*. The researchers confirmed that such biomaterials can exert a prolonged antimicrobial effect even after prolonged exposure. In comparison with the silver NCs or phages alone, the developed biomaterial markedly improved biofilm eradication, especially following 48 h of treatment. Overall, the findings indicate the efficacy of the synergistic phage-NC approach to fight against biofilm-related infections [129].

In a different study, Wang et al. [130] developed a novel bioactive nanoconjugate of drug-loaded liposomes and phages for targeted MDR biofilm eradication in the case of orthopedic infections. The researchers used the phage Sb-1, which has the capacity to degrade the extracellular polymeric substance matrix of bacterial biofilms, for conjugation with antibiotic-loaded liposomes. Following exposure to the biofilms, the phage Sb-1 degraded the extracellular polymeric substance structure, thus increasing the bacterial sensitivity to antibiotics and allowing the antibiotics to penetrate deeply into the biofilm. In addition, low dose of antibiotics was found to be effective in removing MDR bacterial biofilm both in vitro and in vivo. The developed liposome-phage nanoconjugates also efficiently reduced the bacterial load in the infected region and markedly mediated osteomyelitis recovery in the rat prosthetic joint infection model [130]. In another study, Szymczak et al. [45] demonstrated that T7 phages armed with silver NCs were highly effective in controlling bacterial biofilms, in comparison with NCs or phages alone. They identified the novel silver NC-binding peptide and then prepared T7 phages that effectively displayed the peptide on the outer surface of the viral head. The developed silver NC-binding phages efficiently eradicated bacterial biofilms, even at lower concentrations. In addition, these silver NC-binding phages were not toxic to eukaryotic cells [45].

### 4.5. Enhanced Eradication of Bacteria by Using Phage/Nanocarrier Cocktails

Phages are commonly studied as cocktails with antibiotics [35]. These cocktails can improve the eradication of bacteria while decreasing the possibility of resistance development. Phages disturb biofilms of bacteria, enhancing antibiotic penetration as well as efficacy [36], whereas antibiotics weaken bacterial defenses, making them more vulnerable to phage attacks [37]. In a study, Wdowiak et al. [131] studied the synergy between phages and green tea extract-capped silver NCs in combating pathogenic bacteria, including *Salmonella enterica* and MRSA. These NCs showed negligible antiphage actions, which ensured compatibility in phage-NC formulations. In addition, these combinations markedly decreased bacterial counts in a short time; for example, MRSA survival was approximately 30% following incubation with just 0.001 mg/mL of green tea extract-capped silver NCs, while phages and green tea extract-capped silver NCs alone resulted in approximately 70% and 80% survival, respectively. The researchers concluded that green tea extract-capped silver NCs are safe and effective in phage/NC antibacterial formulations with dual amoebicidal and antimicrobial functions for environmental and therapeutic uses [131] (Table 2).

## 5. Challenges and Future Directions

Phage-based therapy can serve as a viable alternative antibacterial strategy in treating infections caused by bacteria that are resistant to commonly used antibiotics [132,133]. In addition, phage-based therapy can also be personalized to each patient as a personalized therapy [134]. Such personalization requires isolation as well as analysis of the clinical pathogenic strains responsible for causing the disease in a given patient, and then comparing them with well-characterized phage bank, which is likely to provide better clinical outcomes [135,136,137].

Although phage therapy offers numerous benefits in terms of selective and enhanced killing of bacteria, phages also suffer from several limitations, including immunological actions, development of bacterial resistance against phage, and secondary infection, as well as co-infection [138]. Thus, there is a growing interest in adding natural products or antibiotics with phages to improve phage effectiveness and decrease the occurrence of phage resistance [139]. The use of phage therapy involving engineered or natural phages often raises various ethical challenges across societal, regulatory, and scientific domains. The major concerns for the development of phage-based therapy include a lack of a predefined regulatory pathway and concerns over intellectual property protection [140]. These concerns further create uncertainty regarding profitability in therapy development and complications in investment recovery. On the other hand, ethical challenges arise from the unpredictable and complex risks of phage therapy, which makes rigorous patient consent procedures crucial owing to the different understanding levels of patients and healthcare providers. Phage therapy also involves concerns regarding the development of ABR because of the horizontal gene transfer capacities of phages, especially during the lysogenic cycle. Therefore, more studies, particularly clinical trials, are required to enhance phage therapy efficacy and ensure reasonable global access to this promising therapy [141].

A key challenge to phage stability is the occurrence of spontaneous mutations in phage stocks during prolonged storage or accumulation during phage production and preparation, which may reduce viral fitness [142]. Although challenging, predicting phage evolution during preparation could aid in developing manufacturing techniques that minimize mutation rates in phage genomes [143]. Due to the high specificity of phage activity, screening large phage collections is necessary to identify phages targeting specific bacterial strains [144]. To enable broader adoption of phage therapy, a rapid, simple, and high-throughput phage screening method should be developed and implemented in phage banks and clinical settings. Furthermore, promoting education about phage therapy is crucial to counter misinformation and enhance its acceptance. Additionally, transparent reporting of the potential, limitations, and challenges associated with its therapeutic application is essential [145].

The interactions between phages and immune cells are yet to be fully understood. Phages are known to interact indirectly or directly with the host’s adaptive and innate immune systems. Nonetheless, the exact mechanism and main immunomodulatory properties are still largely elusive. Extreme production of neutralizing antibodies could be a major challenge in phage therapy, as these antibodies can cause phage clearance prior to the elimination of pathogens [30]. Therefore, a balance is required between immune activation, host immunological status, the dose, and administration routes [26,146]. The usage of bioinformatics tools is crucial in forecasting, phage efficacy, phage, as well as pathogen binding sites. Bioinformatics tools are also important to correctly define phages with precise detection as well as killing capacity against ABR pathogens. Furthermore, bioinformatics and whole-genome sequencing play an important role in various studies regarding phage therapy to tackle any limitations in phage therapy against ABR pathogens [145].

The incorporation of phages with nanotherapeutic systems offers numerous advantages to selectively eradicate pathogenic bacteria; however, still there are significant limitations and challenges in their clinical applications. More studies are required focusing on safety concerns to make sure that phages conjugated with engineered NCs do not generate systemic side effects, and optimization of their efficacy against a wide range of bacteria. Furthermore, more studies are also required to evaluate the controlled-release property, potential risks, metabolic responses, targeting efficiency, and stability of phage-guided NCs. In terms of clinical uses, standardization of manufacturing methods and scalability for large-scale production need to be carefully considered. The effects of the combinations of phage-guided NCs with various other therapies, including photodynamic therapy and immunotherapy, can also be explored to achieve a multidimensional therapeutic outcome. In addition, interdisciplinary collaborations between materials science, immunology, microbiology, and artificial intelligence are important to mediate the commercialization of phage-guided NCs and fast-track the translation from bench to bedside [96]. Phages are genetically modifiable supra-macromolecules. Phage hybridization with inorganic compounds is likely to offer new properties to phages and might be a potential approach for the development of bioinorganic carriers [147].

Although efficacy in experimental findings and animal models indicates potential for safety of human phage-based therapy, there are still major challenges that need to be addressed before standardization of phage therapy for human use, such as potential human immune response induced by phage-based therapy, the pharmacokinetics of phage, and the development of phage-resistant bacteria [148]. On the other hand, NCs have a tendency to form aggregates because of high surface energy. Drying, synthesis, and forces like capillary and Van der Waals forces affect this aggregation [149,150]. Uncontrolled aggregation of NCs is likely to exert negative effects and needs to be avoided. In order to avoid aggregation-related problems, NCs ought to possess excess negative surface charges predominantly in the fasting state and exhibit steric hindrance via surface decoration with anionic surfactants, citrate, and large polymeric chains (including polyvinylpyrrolidone and polyethylene glycol) [151].

## 6. Conclusions

The emergence of MDR bacteria is causing a major public health threat that requires urgent multidisciplinary approaches to develop novel control measures. ABR-associated deaths, because of infections not responding to antibiotics, are becoming an alarming crisis globally, where phage-guided NCs might serve as a game-changing therapy. The integration of phages with NCs can be useful to effectively treat a wide range of infections caused by MDR bacteria. Indeed, phage encapsulation provides enhanced bioavailability, targeted delivery, greater stability, evasion of rapid clearance from the body, and avoidance of loss of activity. Therefore, it is crucial to develop new types of NCs for the delivery of phages by combining the benefits of natural and synthetic polymers. In addition, ameliorated manufacturing methods are required to ensure the scale-up and reproducibility of phage-based NCs to meet industrial manufacturing requirements. More studies, particularly clinical studies, are also required so that phage-guided NCs can see faster progress and wider clinical success. Major challenges also need to be addressed before standardization of phage therapy for human use, including the potential for human immune response induced by phage-based therapy, the complex pharmacokinetics of phages, and the development of phage-resistant bacteria, which can eventually limit the efficacy of phage-based therapy over time. Moreover, innovative strategies, including artificial intelligence-enhanced structural prediction, and bioengineering strategies, including CRISPR-Cas, are also required to improve phage therapies.

## Figures and Tables

**Table 1:** Comparison of antibiotics and phage-based therapies.

Features	Antibiotic Therapy	Phage Therapy	References
Antimicrobial spectrum	Broad-spectrum, thus affects more than the targeted organism	Selectively infect and eliminate bacteria with a high degree of specificity.	[17]
Disruption of beneficial microbiota	Disrupts the balance of the host’s beneficial microbiome, resulting in dysbiosis and potential secondary infections	Minimal impact on the beneficial microbiome	[18,19]
Dosing	Continuous dosing is required to maintain therapeutic levels in the body	Self-amplification in target bacteria after initial administration and enhanced concentration at the target site, therefore, reduces the frequency of administration	[20,21]
Anti-biofilm activity	Ineffective/less effective	Effectively penetrates and destroys biofilms	[22]
Resistance Development	More recurrently used to treat infections; therefore, there is an increased occurrence of a wide range of bacteria developing resistance	Phages co-evolve with bacteria, therefore limited chances of developing resistance	[23,24]
Side effects	Numerous side effects are observed	Side effects related to phage therapy have rarely been reported	[25]
Impact on the immune system	Directly affects the immune system via various immunomodulatory antibiotics	Repeated administration of phages may result in the development of anti-phage antibodies that can neutralize therapeutic phages	[26,27]
Regulatory pathway	Standardized	Complex	[28,29]
Discovery process	Slow	Rapid	[30,31]
Storage stability	Typically show higher and more consistent long-term storage stability	Exhibit variable stability depending on the storage method and phage type	[32]

**Table 2:** A summary of a range of preclinical trials involving phage-guided nanotherapeutic systems.

Phage-Guided Nanotherapeutic Systems	Target Pathogens	Study Outcomes	References
BK510 commercial phage endolysins conjugated with silver nanocapsules (NCs)	*Enterococcus faecium*, *Staphylococcus aureus*, *Klebsiella pneumoniae*, *Acinetobacter baumannii*, *Pseudomonas aeruginosa*, and *Enterobacter* species	Use of the conjugates showed a higher inhibitory effect as compared to silver NCs in over 65% of the Gram-negative bacteria.	[107]
Phage-mimicking antibacterial NCs composed of silver-coated gold nanospheres	*Corynebacterium striatum*, *Enterococcus faecalis*, *Pseudomonas aeruginosa*, and *Staphylococcus aureus*	The bacterial growth of these 4 bacteria was delayed (by up to 5 h) and suppressed (21% to 90%).	[10]
Phage K incorporated poly(N-isopropylacrylamide) nanospheres copolymerized with allylamine	Methicillin-resistant *Staphylococcus aureus*	The developed phage incorporated Poly(N-isopropylacrylamide) nanospheres copolymerized with allylamine successfully lysed *Staphylococcus aureus* at 37 °C, while the growth of bacteria remained unchanged at 25 °C, therefore providing a thermally triggered phage release.	[90]
Polyvinyl alcohol, honey, and chitosan nanofibers were electrospun and loaded with bee venom, propolis and/or phage	*Staphylococcus aureus*, methicillin-resistant *Staphylococcus aureus*, *Escherichia coli*, multidrug-resistant *Pseudomonas aeruginosa*	Among various nanofiber formulations, the combination of polyvinyl alcohol, honey, and chitosan-bee venom/phage exhibited the highest antibacterial efficacy against both Gram-positive and Gram-negative bacteria, achieving near-complete elimination of *Pseudomonas aeruginosa*.	[113]
Phage K-loaded nano-emulsion	*Staphylococcus aureus* strains H560, H325, and Btn766	Phage-loaded nano-emulsion formulations demonstrated superior antibacterial activity compared to freely suspended phages. Additionally, these nano-emulsions achieved rapid and complete eradication of three distinct *Staphylococcus aureus* strains.	[117]
Engineered Mesoporous bioactive glass nanoparticles (MBGNs) by utilizing phage PFPV25.1	*Salmonella* Typhimurium strain LT2	MBGNs were modified with amine groups to improve the affinity between phages and the surfaces of MBGNs. As compared to non-functionalized MBGNs, an increased level of phages was found to be bound onto amine-functionalized MBGNs. In addition, because of this increased phage binding, amine-functionalized MBGNs showed higher antibacterial properties as compared to phage-bound MBGNs.	[120]
Combination of 3 phages (ZCPA5, ZCSS1, and ZCSE6) with 3 green synthesized NCs including silver-chitosan-NCs, pH-sensitive chitosan-NCs, and propolis-chitosan-NCs	*Pseudomonas aeruginosa*, *Staphylococcus sciuri*, and *Salmonella* Typhimurium	Among the NCs, silver-chitosan-NCs showed higher bactericidal properties in combination with phages. In addition, a substantial killing capacity was exhibited by silver-chitosan-NCs (16.5–30.1 μg/mL) in combination with phages.	[121]
Engineered T7 phages armed with silver NCs	*Escherichia coli*	In comparison with the silver NCs or phages alone, the developed biomaterial markedly improved biofilm eradication, especially following 48 h of treatment.	[129]
Novel bioactive nanoconjugate of antibiotic-loaded liposomes and phage Sb-1	Methicillin-resistant *Staphylococcus aureus*	Upon exposure to biofilms, the Sb-1 phage disrupted the extracellular polymeric substance structure, enhancing bacterial susceptibility to antibiotics and facilitating deeper antibiotic penetration into the biofilm. Additionally, the liposome-phage nanoconjugates effectively reduced bacterial load in the infected area and significantly promoted recovery from osteomyelitis in a rat model of prosthetic joint infection.	[130]
T7 phages armed with silver NCs	*Escherichia coli*	The developed silver NC-binding phages efficiently eradicated bacterial biofilms, even at lower concentrations. In addition, these silver NC-binding phages were not toxic to eukaryotic cells.	[45]

## Data Availability

The data presented in this study are contained within this article.
